# Technical Note on Root Coverage of Lower Anterior Teeth Using a Partially Deepithelialized Connective Tissue Graft (PE-CTG) Aided by a High-Speed Handpiece

**DOI:** 10.1155/2020/8815176

**Published:** 2020-08-31

**Authors:** Kyeong-Ok Lim, Byung-Ock Kim, Won-Pyo Lee

**Affiliations:** Department of Periodontology, School of Dentistry, Chosun University, 309 Pilmun-daero, Dong-gu, Gwangju 61452, Republic of Korea

## Abstract

Root coverage in the mandibular anterior region is challenging because of a thin gingival biotype, shallow vestibule, and high frenum attachment. Several methods have been introduced to predict the root coverage in this area. Stimmelmayr proposed a method of performing root coverage using a combination epithelialized-subepithelial connective tissue graft (CTG). However, it is difficult to precisely acquire connective tissue according to this method. Therefore, in this case report, we would like to introduce a technique to harvest a partially deepithelialized CTG (PE-CTG) aided by a high-speed handpiece, which helps in procuring the graft easily and quickly. This method could lower the patient's morbidity at donor sites and enhance the healing process. Additionally, it could increase the amount of keratinized gingiva in the mandibular anterior region without reducing the vestibular depth. Therefore, PE-CTG using a high-speed handpiece can be a promising treatment option for the root coverage of the mandibular anterior teeth.

## 1. Introduction

Gingival recession refers to the apical migration of the gingival margin from the cementoenamel junction (CEJ), resulting in root exposure [1]. In clinical situations, gingival recession is commonly observed after orthodontic treatment, especially in the mandibular anterior region. If the tooth is positioned outside the alveolar bone during or after orthodontic movement, it may cause dehiscence of the alveolar bone and thinning of the volume of the facial soft tissue [2]. Since the gingiva is thinner in the mandibular anterior region than in other regions, gingival recession often occurs. Additionally, orthodontic treatment can alter oral environment [3]. Periodontal procedures have been shown to be effective in the resolution of deep gingival defects over a long follow-up period [4]. Therefore, the possibility of gingival recession before and after orthodontic treatment should be predicted and treatment should be considered.

The mandibular anterior region has a thin gingival biotype and reduced width of keratinized tissue. During a coronally positioned flap (CPF) surgery, passive surgical positioning of the flap is difficult because of the shallow labial vestibule, high frenum attachment, and mentalis muscle activity. Therefore, it is difficult to achieve complete root coverage (CRC), and recurrence after surgery is high [5].

To address these issues related to the characteristics of the mandibular anterior region, Stimmelmayr et al. developed the tunnel technique with a combination epithelialized-subepithelial CTG [6]. Partly deepithelialized connective tissue except for the designed epithelial portion equal to the size of the gingival recession defect is grafted through a tunnel flap onto the mandibular anterior region. However, this method requires technique-sensitive skills to harvest the connective tissue from the palate.

Therefore, in this case, we introduce a technique to harvest a partially deepithelialized connective tissue graft (PE-CTG) aided by a high-speed handpiece, making root coverage easier and quicker in the mandibular anterior region.

## 2. Case Report

**Figure 1:**
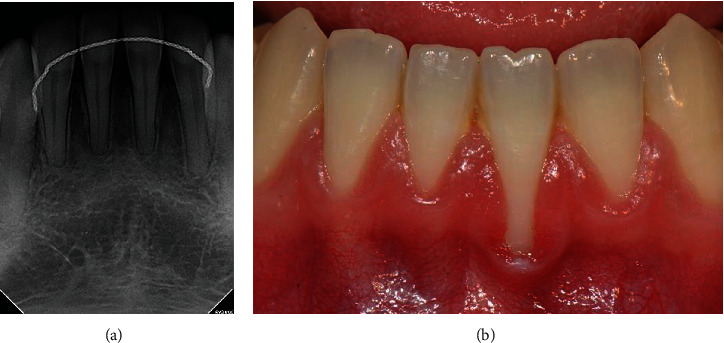
A 22-year-old man with gingival recession in the mandibular left central incisor. (a) Periapical radiograph. (b) Intraoral photograph of the labial side.

A healthy 22-year-old man presented with a chief complaint of gingival recession after orthodontic treatment. When measured with a periodontal probe (Hu-Friedy Inc., Chicago, IL, USA), gingival recession with a depth of 6 mm was observed in the left mandibular central incisor. The gingival recession defect was measured to a depth of 6 mm, width of 3 mm, and apicocoronal keratinized tissue width (KTW) of 0 mm. The height of the interdental bone was normal, but loss of the interdental papilla was observed (Figures 1(a) and 1(b)). The defect was diagnosed as Miller's class III recession [7] and Cairo's type 2 recession [8], and partial root coverage was predicted.

### 2.1. Surgical Procedures

**Figure 2:**
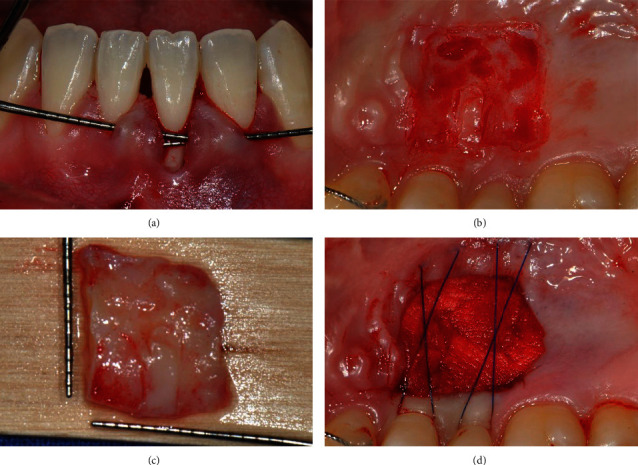
(a) Tunnel preparation. (b) Deepithelialization using a high-speed handpiece at the hard palate. (c) Partially deepithelialized connective tissue graft. (d) Collagen dressing of the donor site.

After local anesthesia was administered using 2% lidocaine with 1 : 100.000 epinephrine (Yuhan Co., Seoul, Korea), all inflammatory tissues were removed using a Gracey curette (LM instruments Oy, Planmeca Group, Parainen, Finland), and root planing was performed on the exposed root surface. As suggested by Allen [9, 10], a sulcular incision was performed on the left central incisor. Thereafter, a supraperiosteal tunnel was made labially from the right central incisor to the left lateral incisor. The tunnel was sufficiently prepared to accommodate a connective tissue. It was ensured that no trauma occurred to the marginal gingiva (Figure 2(a)).

### 2.2. Graft Harvesting

A PE-CTG was obtained from the hard palate of the maxillary left premolar region. It was designed with a 15C blade (KIATO®, Kehr Surgical Private Ltd., Hannover, Germany) according to the shape, size, and position of the gingival recession defect measured previously. Thereafter, approximately 0.5-1 mm of epithelial layer was removed using a high-speed handpiece and a 2 mm-diameter round diamond bur, except for the designed epithelial portion (Figure 2(b)). The border of the epithelialized part was clearly formed so that it could be attached by a butt joint at the recipient site. The marginal part of the connective tissue was removed slightly beyond the border line to avoid leaving the epithelium. The graft was carefully retrieved through a split-thickness flap using a 15C blade (Figure 2(c)). The donor site was dressed with collagen matrix (Collatape®, Zimmer Dental, Mississauga, Canada) and secured with crossed horizontal sling suture technique using 5-0 monofilament suture (Dafilon®, B Braun, Melsungen, Germany) (Figure 2(d)).

### 2.3. Transplantation and Fixation of the Connective Tissue

**Figure 3:**
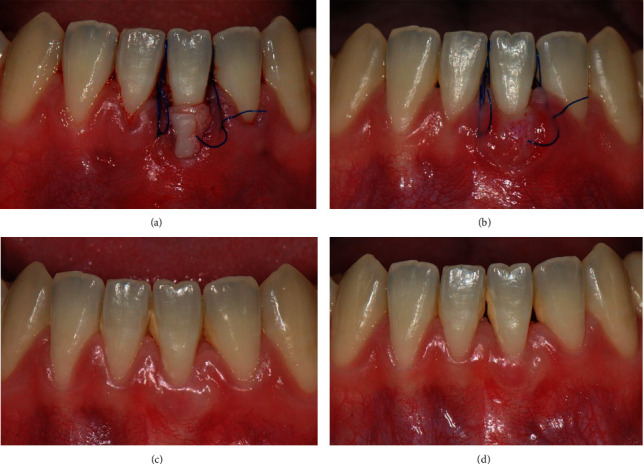
(a) The graft was introduced and secured using a vertical double-crossed suture. (b) Superficial epithelial sloughing and capillary ingrowth were observed at 1 week. (c) Optimal graft incorporation and complete reepithelization were noted at 4 weeks. (d) Complete root coverage and adequate attached gingiva were obtained at 1 year after surgery.

Through the sulcular access of the left central incisor, the graft was introduced into the tunnel, and the epithelial portion of the graft was positioned on the exposed root. Subsequently, the graft site on the left and right central incisors was secured using 5-0 monofilament suture (Dafilon®, B Braun, Melsungen, Germany) with the vertical double-crossed suture technique proposed by Zuhr et al. [11] (Figure 3(a)).

The patient was prescribed ibuprofen 600 mg twice a day for 7 days. He was instructed to avoid mechanical home care at the surgical site for 6 weeks and instead was advised to rinse with a 0.1% chlorhexidine digluconate solution (Hexomedine solution, Bukwang Pharm. Co., Seoul, Korea) twice daily. The sutures at the donor site were removed after 1 week, and those at the recipient site were removed 2 weeks after surgery.

### 2.4. Wound Healing

Initially, it was observed that the epithelial layer was sloughing, and revascularization was observed underneath the tissue (Figure 3(b)). However, 1 month after surgery, the graft was completely reepithelized and assimilated into the surroundings with harmonious color and shape (Figure 3(c)). The height of the gingival margin and the location of the mucogingival junction (MGJ) were also in harmony with the surrounding tissue. In this case, CRC was achieved by covering the exposed root surface up to the CEJ. Furthermore, the apicocoronal KTW was measured at 4 mm after the surgery, indicating an increase in the keratinized gingival width. At 1 year postoperatively, no remarkable inflammation was observed, and the transplanted tissue was maintained (Figure 3(d)).

## 3. Discussion

Various factors may contribute to the low success rates of root coverage in the mandibular anterior region. Important factors among them may be related to the unique anatomical elements in this area, such as thin biotype, prominent roots, high frenum attachments, shallow vestibule, mentalis muscle activity, and crowding of teeth. Harris et al. reported that the mean root coverage (MRC) of the mandibular anterior region was 95.7%, which was lower than other regions with 97.1 to 100% coverage, except the maxillary posterior region [12]. Another study reported that MRC with CPF and CTG was 90.9% when the gingival recession depth was less than 3 mm, but when the depth was 3 mm or more, it significantly decreased to 68.4% [13]. Therefore, as the gingival recession depth increases, it is necessary to completely cover the graft with a flap to increase its survival rate. In such cases, an increase in the coronal advancement of the flap is unavoidable. However, if the coronal positioning of the flap is increased in the mandibular anterior region with a shallow vestibule and high frenum attachment, strong retraction forces will occur in the flap after surgery. This would make it difficult to achieve successful root coverage and result in a high relapse rate. Therefore, excessive repositioning of the flap should be avoided, especially in the mandibular anterior region.

In cases where the recession defect is large, such as the gingival recession defect of 6 mm in our case, reducing the coronal positioning of the flap to prevent shallow vestibular depth or displacement of the MGJ results in graft exposure. Han et al. performed a root coverage surgery by intentionally exposing the graft by 1-2 mm [14]. According to the results, it was observed that the CTG exposed by approximately 1-2 mm did not show statistically significant difference in root coverage. Rather, intentionally exposing the graft may secure more keratinized gingiva and prevent displacement of the MGJ. However, the success rate of root coverage cannot be guaranteed when a graft is exposed by more than 1-2 mm. This is because greater exposure of the graft will result in lesser blood supply from the flap and higher chances of necrosis. These problems make it difficult to achieve successful root coverage [10]. According to Yotnuengnit et al., the root coverage outcomes were closely related to the ratio of graft tissue area (GTA) and visible denuded area (VDA) [15]. In particular, the GTA : VDA should be at least 11 : 1 in order to obtain complete root coverage. However, if the recession defect is as large as 6 mm, as in this case, a graft of a fairly large size must be obtained, which will affect the patient's morbidity.

**Figure 4:**
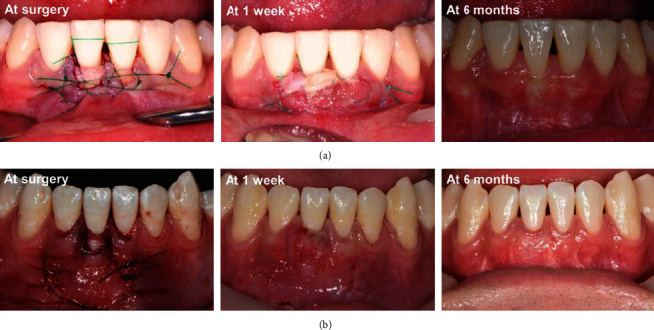
(a) In a large recession defect, when the connective tissue is exposed without leaving the epithelialized portion, necrosis of the graft is observed at 1 week after the surgery. Complete coverage did not occur after 6 months. (b) When PE-CTG was performed in a case with a gingival recession defect 5 mm in depth, the epithelialized portion was sloughed and capillary ingrowth was observed in the underlying connective tissue 1 week after the surgery. Even after 6 months of surgery, complete root coverage was maintained.

In order to compensate for this problem, Stimmelmayr et al. performed root coverage using partially epithelialized connective tissue so that the exposed root surface could be covered by the epithelial portion. This technique protects the underlying connective tissue while the epithelialized part of the graft undergoes sloughing, thereby, reducing the risk of necrosis of the connective tissue, increasing capillary ingrowth into the connective tissue, and allowing time for epithelialization [6]. According to our clinical experience, in the case of large recession defects, if the epithelial portion remained above the graft, the upper portion of the graft underwent necrosis, and sufficient coverage was not achieved. However, when the graft containing the epithelialized portion was transplanted, the epithelialized part disappeared due to sloughing, but it was observed that capillary ingrowth occurred in the underlying connective tissue and its volume was maintained (Figure 4).

Various factors can be considered to measure the donor site morbidity [16, 17]. According to several studies, the main cause of postoperative pain at the donor site was sloughing of the epithelium during the healing process [18–20]. In general, it is known that harvesting the graft in the form of FGG has a greater donor site morbidity than that in CTG [21]. Zucchelli et al. compared the degree of pain on the palate after surgery according to the graft harvesting method [22]. In case of the trap-door approach, the secondary healing process required a significantly higher dose of analgesic since the epithelial layer was necrotic. However, when the connective tissue was obtained with the trap-door approach and primary healing occurred without epithelial sloughing, there was no significant difference in the analgesic dose compared to that of the FGG approach. It can be considered that the discomfort of the patient does not increase if the connective tissue is obtained in the form of FGG and secondary healing is guided. In addition, according to Wessel et al., when the graft was obtained in the form of FGG, the degree of pain on the third day was greater than that with CTG, but there was no significant difference after 3 weeks [16].

The size of the graft obtained from the donor site is also associated with the donor site morbidity [17, 20, 23]. As explained earlier, Yotnuengnit et al. stated that the size of the graft should be at least 11 times larger than the VDA [15]. However, if the recession defect is large, as in this case, a significantly larger graft is needed. In this case, the GTA and VDA were approximately 122 mm^2^ and 15 mm^2^, respectively. Therefore, the GTA : VDA was approximately 7.13, and a complete root coverage was obtained even with CTGs much smaller than the ratio suggested by Yotnuengnit et al. This suggests that the size of the graft can be decreased to reduce the donor site morbidity while simultaneously achieving predictable results.

The lamina propria induces connective tissue differentiation into the epithelium [23]. According to Cho et al., the lamina propria histologically exists beneath the keratinized stratified squamous epithelium and has an average thickness of 1 mm [24]. The epithelium has an average thickness of 0.3 mm. If a graft is obtained after partial deepithelialization to a depth of approximately 0.5-1 mm, the lamina propria remains above the graft. This contributes to the formation of keratinized gingiva after the epithelialized portion has sloughed. If the deepithelialization is performed with a bur, the epithelial layer can be removed uniformly and easily. The CTG is harvested with a thickness of 2 mm, so that the connective tissue layer including the periosteum can be left above the donor site bone, thereby preventing bone exposure. This can significantly reduce the pain caused by bone exposure at the donor site and shorten the healing period. Therefore, the connective tissue of appropriate thickness can be acquired even from a thin palate.

Various instruments can be used to obtain the graft [25]. Stimmelmayr et al. used the combination epithelialized-subepithelial CTG technique to leave an epithelial layer equal to the size of the defect above the graft using a blade when harvesting the connective tissue [6]. However, secondary healing at the donor site occurs according to the size of the defect, accompanied by epithelial sloughing and increased pain. Moreover, leaving the epithelial layer in the shape of the defect and raising a partial thickness flap increases the technique sensitivity and is likely to retrieve an unwanted graft. Therefore, in this case, a high-speed handpiece and a round diamond bur were used to obtain a partially deepithelialized graft. Harvesting the graft in the form of FGG has the advantage of being easier and faster than Stimmelmayr et al.'s method. Moreover, the epithelialized portion can be formed precisely by designing the graft. The additional advantage of this method is that the patient's palatal discomfort is not high after the surgery.

This report had a few limitations. First, this case report involved a gingival defect in a single tooth. It is necessary to conduct similar studies with a large sample size in the future. Further, the follow-up period was short; therefore, long-term observation of such cases is necessary.

## 4. Conclusion

Performing root coverage in the mandibular anterior region, using a high-speed handpiece to obtain PE-CTG, is an easier and faster technique with predictable results. Particularly, in case of a recession defect of 3 mm or more, it can be selected as the highest priority treatment option without resulting in a shallow vestibule or displaced MGJ.

## Data Availability

No data were used to support this study.
